# Immune Evasion Strategies of Ranaviruses and Innate Immune Responses to These Emerging Pathogens

**DOI:** 10.3390/v4071075

**Published:** 2012-06-28

**Authors:** Leon Grayfer, Francisco De Jesús Andino, Guangchun Chen, Gregory V. Chinchar, Jacques Robert

**Affiliations:** 1 Department of Microbiology and Immunology, University of Rochester Medical Center, Rochester, NY 14642, USA; Email: Leon_Grayfer@urmc.rochester.edu (L.G.); Francisco_Dejesus@urmc.rochester.edu (F.D.J.A.); gchen1104@aggiemail.usu.edu (G.C.); 2 Department of Microbiology, University of Mississippi Medical Center, Jackson, MS 39216, USA; Email: vchinchar@umc.edu

**Keywords:** Iridovirus, ranavirus, FV3, frog virus 3, innate immunity, macrophage, anti-viral, immune-evasion, cytokines, inflammation

## Abstract

Ranaviruses (RV, *Iridoviridae*) are large double-stranded DNA viruses that infect fish, amphibians and reptiles. For ecological and commercial reasons, considerable attention has been drawn to the increasing prevalence of ranaviral infections of wild populations and in aquacultural settings. Importantly, RVs appear to be capable of crossing species barriers of numerous poikilotherms, suggesting that these pathogens possess a broad host range and potent immune evasion mechanisms. Indeed, while some of the 95–100 predicted ranavirus genes encode putative evasion proteins (e.g., vIFα, vCARD), roughly two-thirds of them do not share significant sequence identity with known viral or eukaryotic genes. Accordingly, the investigation of ranaviral virulence and immune evasion strategies is promising for elucidating potential antiviral targets. In this regard, recombination-based technologies are being employed to knock out gene candidates in the best-characterized RV member, Frog Virus (FV3). Concurrently, by using animal infection models with extensively characterized immune systems, such as the African clawed frog, *Xenopus laevis*, it is becoming evident that components of innate immunity are at the forefront of virus-host interactions. For example, cells of the macrophage lineage represent important combatants of RV infections while themselves serving as targets for viral infection, maintenance and possibly dissemination. This review focuses on the recent advances in the understanding of the RV immune evasion strategies with emphasis on the roles of the innate immune system in ranaviral infections.

##  Abbreviations

[**ATV**]: * Ambystoma tiginum* virus
[**BIV**]: Bohle Iridovirus
[**CARD**]: caspase activation and recruitment domain
[**CCV**]: channel catfish herpes virus
[**DE**]: delayed early genes
[**EHNV**]: epizootic haematopoietic necrosis virus
[**eIF2α**]: eukaryotic translation initiation factor 2 alpha
[**FV3**]: frog virus 3
[**HIV**]: human immunodeficiency virus
[**IE**]: immediate early genes
[**IFNγ**]: interferon gamma
[**IL-1β**]: interleukin-1 beta
[**IRF**]: interferon regulatory factor
[**L**]: late genes
[**MAPK**]: mitogen activated protein kinase
** [MX1**]: Myxovirus-resistance1
[**ORF**]: open reading frame
[**PKR**]: RNA-dependent protein kinase
[**PL**]: peritoneal leukocyte
[**RCV-Z**]: *Rana* (*Lithobates*)* catesbeiana* Virus Z
[**RV**]: ranavirus
[**SGIV**]: Singapore Grouper Iridovirus
[**TGIV**]: Taiwan Grouper Iridovirus
**[TNFα**]: tumor necrosis factor alpha
[**vIFα**]: viral translation initiation factor-alpha homolog

## 1. Introduction

Over the last two decades it has become increasingly apparent that amphibian species are facing a serious threat of extinction [1], where roughly one-third (32%) of the 6593 amphibian species are diminishing as a result of complex, as of yet poorly understood causes. A number of possible escalating factors have been attributed to these die-offs, including destruction of habitats, increased levels of pollution, changes in climate as well as increasing ultraviolet irradiation [2,3]. While these may be underlining mechanisms, there is also a prevailing theory that the increasing amphibian declines stem from compromised immune systems of these animals coupled with elevated pathogenic threats [2,3], undoubtedly resulting from one or a combination of the above. 

Until recently, it was believed that viral infections were a secondary contributing factor in these die-offs. However, currently members of the family *Iridoviridae* and particularly the genus *Ranavirus* (RVs, family *Iridoviridae*) have gained attention due to the dramatic rise in the prevalence of RV infections across pokilothermic species. In fact, ranaviruses are now considered the second most common infectious agent plaguing wild and cultured amphibian species [2,3], with half of the amphibian deaths in United States between 1996 and 2001 attributed to ranaviral infections [4]. 

Ranaviruses are icosahedral, double-stranded DNA viruses with large genomes, ranging between 105 and 140 kilobase pairs in size. Specifically, members of the family *Iridoviridae* are known to infect three classes of ectothermic vertebrates: amphibians, bony fishes (teleosts) and reptiles [5]. To date three RV species that infect amphibians have been identified and grouped according to genetic and ecological parameters [6]. Amongst these, Bohle Iridovirus (BIV) infects Australian frogs and has so far remained confined to this region of the world. *Ambystoma tiginum* virus (ATV) infects salamanders and is primarily localized to United States and Canada. In contrast, the Frog Virus 3 (FV3), initially isolated from the leopard frog, *Rana* (*Lithobates*)* pipiens*, has been recognized worldwide as an amphibian pathogen. With a rapid increases in the prevalence and spread to multiple amphibian species, FV3 is believed to be a potential global threat to amphibian populations [7]. Although, FV3 is the greatest threat to pokilothermic vertebrates, information gained from studies dealing with the other two RV species and indeed from other members of the family *Iridoviridae* (generically referred to as “iridovirids” to distinguish them from members of the genus *Iridovirus*) should be recapitulated in order to better understand the mechanisms of infection and immunity within this family.

It is also becoming evident that RVs likely possess a plethora of immune evasion and host modulation mechanisms. A closer examination of the relationships between these viruses and their host immune systems is clearly warranted in light of increasing ranaviral prevalence and the potentially declining immune capacities of the ectothermic species that they infect. As compared to mammals, lower vertebrates such as those infected by iridovirids, possess functional but relatively less effective adaptive immune systems, with fewer antibody classes, poorer T lymphocyte expansion and generally less developed immunological memory responses (reviewed in reference [8]). Accordingly these organisms likely rely more heavily on innate immune components for pathogen clearance. In turn, cells of the macrophage lineage are indispensable for innate immune responses. In mammals, macrophages are long-lived, terminally differentiated cells of myeloid origin that exhibit limited proliferation capabilities and a high level of heterogeneity [9]. During certain viral infections, distinct macrophage subsets participate in anti-viral responses while in other instances mononuclear phagocytes may become productively infected and serve as long-term viral reservoirs and agents of viral dissemination. For example, during HIV infections macrophages are hijacked by the virus, store large numbers of virions and facilitate cell-to-cell spread of HIV [10,11,12]. Conversely, as sentinels of the immune system, macrophages recognize viral infections through a repertoire of pattern recognition receptors [13,14,15] and facilitate viral clearance by producing an array of bioactive molecules. Thus macrophages function in contrasting ways to either perpetuate virus replication or to eliminate it. 

This review coalesces the current knowledge of the roles of innate immune components in ranaviral infections as well as recent advances in the understanding of ranavirus immune evasion strategies.

## 2. The Role of Myeloid Cells in Ranaviral Infections

### 2.1. Mammalian Models of Ranaviral Infections

Interactions between Frog Virus 3 and cells of the innate immune system were first described over 30 years ago [16,17,18]. Employing a rat hepatitis model of FV3 infections, it was demonstrated that liver macrophages (Kupffer cells) of virally-infected animals were primary targets of FV3 [16]. The necrotic death of these cells was linked to lack of hepatic clearance and subsequent toxicity leading to severe hepatitis and animal deaths [16]. Interestingly, elevated production of leukotrienes by FV3-infected rat Kupffer cells proved to be a contributing factor in this hepatitis progression [19]. Rats infected with this pathogen exhibited elevated systemic levels of N-Acetyl leukotriene E4 while the administration of specific chemical inhibitors targeting leukotriene synthesis drastically ablated FV3-induced hepatocyte damage [19]. Furthermore, FV3-infected human Kupffer cells retained the capability to adhere to opsonized sheep red blood cells and initiate pseudopodia formation, but were however not capable of completing phagocytic events [17]. 

Although mammalian cells grow at 37 °C and are thus not permissive to ranaviral replication [20], these earlier studies provide information regarding the FV3 tropism and suggest that mononuclear phagocytes may represent central cellular targets of RV infections, presumably due to their high phagocytic and endocytic activities. Accordingly, shortly after infection viral particles have been detected in phagocytic vacuoles and endocytic compartments [21]. Approximately a quarter of attached virions exhibited viral-host membrane fusion events and viral core material were released into cell cytoplasm [21]. These results suggest that while ranaviruses has evolved to thrive in ectothermic vertebrates, the viral cell entry strategies are broad enough to allow invasion of target cell types of evolutionarily distant organisms, such as the murine macrophage. Accordingly, myeloid cells may be viral targets precisely because they actively take up extracellular particles (including virions) through a plethora of wide-spectrum endocytic/phagocytic receptors. This in turn suggests a potential RV infection strategy and perhaps explains why FV3 has been so successful at crossing host species boundaries. Additionally, since FV3 is unable to replicate at non-permissive mammalian body temperatures, this implies that FV3 does not elicit the observed pathogenic effects through newly synthesized viral proteins but rather through one or more virion-associated proteins. Furthermore, factors responsible for the inhibition of host RNA, DNA and protein synthesis [22] can be solubilized from the viral particles [23], and are sufficient for inhibiting host cell nucleic acid synthesis [20]. 

### 2.2. Xenopus Macrophage Model of Ranaviral Infections

The above observations suggest the involvement of myeloid cells in FV3 infections while our investigations performed using FV3 infections of the *Xenopus* model are consistent with this notion. Our group has established a reliable infection model system using FV3 infections of the African clawed frog, *Xenopus laevis* in order to investigate the ranavirus—host immune system interface. Our earlier work revealed that viral DNA could be detected in frogs, months subsequent to their infection and could also be found in apparently healthy animals that were not experimentally infected, suggesting that FV3 might establish either quiescent or persistent infections in amphibians [24]. Furthermore, we have observed that FV3 actively infects frog peritoneal leukocytes (PLs), wherein the virus persisted and underwent gene transcription for up to 12 days post infections [24]. These studies implicated an immune cell subset present in the PL population as a potential vehicle for viral dissemination or as a viral reservoir. 

**Figure 1:**
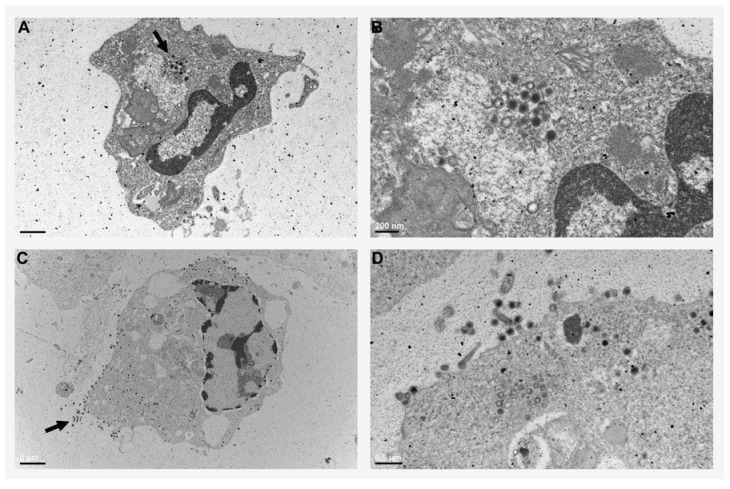
**Electron micrographs of peritoneal macrophages from FV3-infected *Xenopus laevis* adults.** Peritoneal leukocytes were collected from frogs 2 days subsequent to infection with 5 × 10^6^ PFU of FV3, processed and visualized under a Hitachi 7650 TEM. (**A**) Mononucleated macrophage-like cells with an intracellular pool of FV3 virions (arrow, scale bar: 2 μm). (**B**) A magnified image of the intracellular macrophage pool of FV3 virions (scale bar: 200 nm). (**C**) A macrophage-like cell shedding viral particles (arrow, scale bar: 2 μm). (**D**) A magnified image of a virus-shedding macrophage (scale bar: 0.5 μm).

Indeed, subsequent transmission electron microscopy analysis of FV3-infected *Xenopus* peritoneal leukocytes clearly revealed icosahedral virion particles in macrophage-like cells (Figure 1). Viral particles accumulated as intracellular pools in cytosolic vacuoles of these cells (Figure 1A,B), suggesting that FV3 might employ phagocytes as a means of storage and dissemination, reminiscent of the HIV-macrophage relationship. Notably, some of the FV3-infected *Xenopus* peritoneal macrophages also shed numerous FV3 particles (Figure 1C,D), confirming that these phagocytes may serve as both viral stores as well as effective means of viral dissemination.

In subsequent work, we observed that FV3 infections of adult frogs resulted in increased recruitment of leukocytes to the peritoneum (sites of FV3 inoculation in these studies) of infected adult frogs [25]. Of note, a large proportion of these PLs comprised of monocyte-macrophage like cells and FV3 infections also elicited significantly increased expression of the macrophage pro-inflammatory genes (TNFα and IL-1β) in the PL populations [25]. Interestingly, while FV3 DNA could be detected by PCR in PLs of infected frogs up to 21 days post infection, the expression of the FV3 early and late genes was detected at 6 but not 15 or 21 days post infection [25]. This result suggests that FV3 virions remain cell-associated within macrophages for an extended period of time after infection. Since the frog kidney appears to be a central site for FV3 replication, we also examined the persistence and viral gene expression in this tissue in comparison to peritoneal leukocytes. Viral DNA was detected in kidneys of some but not all infected animals for up to 14 days post infection, while the viral gene expression was seen in some, but not all animals up to 9 days post FV3 infection. Conversely, viral DNA was detected in PLs of some frogs up to 20 days post infection whereas active viral gene expression was not detected in these immune cells after 6 days [25]. This suggests that FV3 can adopt an intracellular quiescent state at later times during infection. Together our past and current work suggests that the frog macrophages are targets of the FV3 infection and possibly serve as reservoirs or vehicles of dissemination while also playing a protective role in viral clearance. Indeed, *in vitro* infected frog PLs may harbor FV3 DNA without detectable viral gene expression for as long as several months after infection (unpublished observations). As such *X. laevis* monocytic cells may prove to be an excellent *in vitro* model for studying viral persistence. 

### 2.3. Macrophage Involvement in Iridovirid Infections of Other Poikilotherms

As emphasized above, there is growing evidence that ranaviruses and in particular FV3 target and utilize myeloid cells as a part of their infection strategy. It is probable that like many other pathogens, ranaviruses overcome macrophage-triggered antimicrobial mechanisms, at which point the macrophages would become agents of dissemination and sources of persistence. Interestingly, an *Iridovirus*-like agent has been reported to infect sheatfish kidney macrophages and down-regulate the production of phorbol myristate acetate-elicited reactive oxygen intermediates *in vitro* [26]. Likewise, groupers infected with Taiwan Grouper Iridovirus (TGIV) characteristically exhibited increased numbers of acid phosphatase positive and highly phagocytic basophilic and eosinophilic cells [27]. These cells appeared to be monocytic in origin and contained TGIV DNA within the nucleus at early times after infection [27]. Conversely these cells displayed both nuclear and cytosolic staining for TGIV and lost phagocytic potentials at later times in the infection (4 days post infection) [27]. 

Thus, it would appear that not just FV3, but other iridovirids infect phagocytes and alter their antimicrobial potentials. Further research, using *in vitro* macrophage infection model systems will yield a better understanding of and perhaps provide therapeutic strategies against these infectious agents. 

## 3. Innate Immune Responses to Ranaviral Infections

### 3.1. Antimicrobial Peptide Responses to Ranavirus Infections

Frogs are known to possess a number of skin-derived antimicrobial peptides. Two such peptides, Esculentin-2P (E2P) and Ranatuerin-2P (R2P), identified in the *Rana* (*Lithobates*) *pipiens* species, are thought to function by disrupting pathogen membranes [28]. These antimicrobial peptides were derived from *R. pipiens* skin and assessed for potential antiviral activity against a channel catfish herpesvirus (CCV) and FV3 [28]. While mammalian antimicrobial peptides require longer time durations to confer antimicrobial activity, E2P and R2P inactivated both FV3 and CCV within minutes (as determined by plaque assays) and were capable of producing these effects at temperatures of 0, 18 and 26 °C, suggesting direct viral inactivation rather than inhibition of viral replication [28]. The broad temperature range at which they function (0–26 °C) is likely reflective of these peptides being derived from an ectothermic organism, requiring functionality across a range of temperatures. While CCV is an enveloped virus, FV3 may infect cells as both enveloped and non-enveloped forms. Interestingly, both E2P and R2P were capable of 99% inactivation of CCV at a doses of 50 μM while ten times greater concentrations of each peptide were required to inactivate 90% of FV3 [28]. Presumably the differences in the antimicrobial peptide concentrations needed to inactivate CCV and FV3 stem from the fact that in the case of CCV, the essential membrane being disrupted is the outer envelope while FV3 inhibition might require the disruption of the inner lipid membrane underlining the FV3 capsid, thus requiring relatively higher concentrations of these antimicrobial peptides. 

### 3.2. Pro-inflammatory Responses to Iridovirid Infections

#### 3.2.1. Cell Signaling Pathways Induced by Iridovirids

The Singapore Grouper Iridovirus (SGIV) is an increasing economic concern to the grouper aquacultural industry. This virus encodes 162 putative genes with 62 identified proteins. When a fish cell line (EAGS) was infected with this virus, activation through phosphorylation of the mitogen activated protein kinase (MAPK) p38 was observed within an hour of infection and was predominantly nuclear 24 hours after infection [29]. Conversely, in SGIV-infected EAGS cells, c-Jun N-terminal kinase (JNK) became phosphorylated 2 hours after infection, suggesting a difference in the kinetics of the activation of these two MAPKs in response to infection [29]. Using chemical inhibitors of p38 and JNK, it was shown that SGIV-induced expression of the pro-inflammatory genes interferon regulatory factor-1 (IRF-1), interleukin-8 (IL-8) and tumor necrosis factor-alpha (TNFα) were dependent on JNK signaling while p38 was only responsible for some of the virally induced TNFα gene expression [29]. Interestingly the viral gene expression, protein synthesis and overall titers were not effected by the MAPK chemical inhibition of infected EAGS cells, suggesting that possibly this virus has evolved to deal with this elicitation of the pro-inflammatory response. It is difficult to speculate to this effect in absence of *in vivo* studies or other *in vitro* studies using instead immune cells as infection targets. However, as described below, there is definite evidence suggesting viral induction of pro-inflammatory gene expression such that of TNFα.

#### 3.2.2. *Xenopus* Tadpole and Adult Inflammatory Responses to Ranavirus Infections

The immune responses of *Xenopus* larvae appear to be much less effective at dealing with infections than those of adult frogs. While adults mount rapid innate immune responses followed by relatively potent CD8 T cell and humoral responses, resulting in viral clearance [30], tadpoles exhibit poorer adaptive immune effectors functions, presumably contributing to the observed susceptibility and mortality of tadpoles seen during FV3 infections [31]. However, there are definite adaptive contributors governing immune outcomes of FV3 infection of *X. laevis* tadpoles and adults [30,31,32]. As mentioned above, *Xenopus* (both tadpole and adult) adaptive immune systems are not as effective as those of higher vertebrates. The onset of these responses in the context of viral infection would occur approximately a week after initial inoculation, at which point the virus would be disseminated throughout the organism. Accordingly it can be argued that components of the innate arm of the immune response are pivotal in limiting the initial spread of viral infection and likely indispensable to viral clearance. Despite this, little is known about the roles of innate immunity, especially in *X. laevis* larvae during the early stage of ranaviral infections. Recently our group has embarked on the characterization of the innate immune responses at early stages of FV3 infections. FV3 infections of adult frogs elicited rapid increases in the gene expression of pro-inflammatory cytokines such as tumor necrosis factor alpha (TNF-α) and interleukin-1β (IL-1β) [25], which are known to target macrophage lineage cells and orchestrate robust innate immune processes. We believe that this infection-elicited innate immune response plays a critical role in conferring the FV3 resistance observed in *X. laevis* adults. 

In contrast to adults, *X. laevis* tadpoles are incapable of clearing FV3 infections and generally succumb to them within a month of inoculation. Despite this, we have observed a certain degree of variability with respect to the larval survival times after FV3 infections, suggesting that the tadpole immune systems, though less developed than those of adults, might still confer some antiviral protection (manuscript in review). In a recent effort to better understand the involvement of innate immune components in *Xenopus* tadpole responses to FV3, we assessed the kinetics of pro-inflammatory cytokine gene expression throughout FV3 infection. Our studies focused on examining the expression of the frog cytokines: TNFα, IL-1β, interferon gamma (IFNγ), and a type I IFN-inducible Myxovirus-resistance (MX1) during early phases of FV3 infection (manuscript in review). Notably TNFα and IL-1β are produced in large quantities by macrophage lineage cells [33,34,35,36,37,38,39]. Moreover, these cell types are themselves primary targets of TNFα, IL-1β and IFNγ, and their phagocyte antimicrobial potentials are greatly enhanced upon stimulation with these cytokines [40,41,42,43,44,45,46,47,48]. The involvement of these cytokines in immune responses against viruses has been well documented in several mammalian and fish species [29,49,50,51,52]. We observed that FV3 infection of tadpoles elicited relatively modest (10–100 times lower than adults) and delayed (3 days later than adult frogs) up-regulation of TNFα, IL-1β, IFNγ and MX1 genes in peritoneal leukocytes and in infected tissues of tadpoles. In contrast, the same genes responded more rapidly to heat-killed bacterial stimulation. Interestingly, tadpoles exhibited significantly greater magnitudes of leukocyte recruitment to the peritoneum after i.p. inoculations of FV3, as compared to adults (manuscript in review). While this suggests that the larval susceptibility stems at least in part from poor viral recognition and/or an ineffective innate immune response, it should be underlined that at the mRNA levels, *X. laevis* tadpoles express significantly greater magnitudes of baseline (*i.e.*, constitutive) mRNA levels of TNFα and IL-1β, but not IFNγ than do adults. This is reminiscent of the expression patterns of these respective immune genes in bony fish, where TNFα and IL-1β [46,53,54] exhibit high constitutive expression levels while IFNγ expression is lower and requires induction [44,45,55]. In light of this, it becomes more difficult to make inferences as to the efficacy of tadpole innate responses. Perhaps it is in the best interest of the frog larvae not to exacerbate already high inflammatory cytokine levels while the tissue damage resulting from later stages of FV3 infections does just that, resulting in eventual tadpole mortality from an overactive, rather than an ineffective inflammatory response. As described above, the mice and rats infected with FV3 succumb to its toxic effects as a result of an excessive inflammatory response. Undoubtedly, the extremely large doses of FV3 (2 × 10^7^–4 × 10^8^ plaque forming units) administered to these animals contributed to these toxic effects. In spite of this, it is possible that adult frogs possess more effective control mechanisms of their inflammatory responses than do tadpoles, possibly contributing to the observed susceptibility differences. 

**Figure 2:**
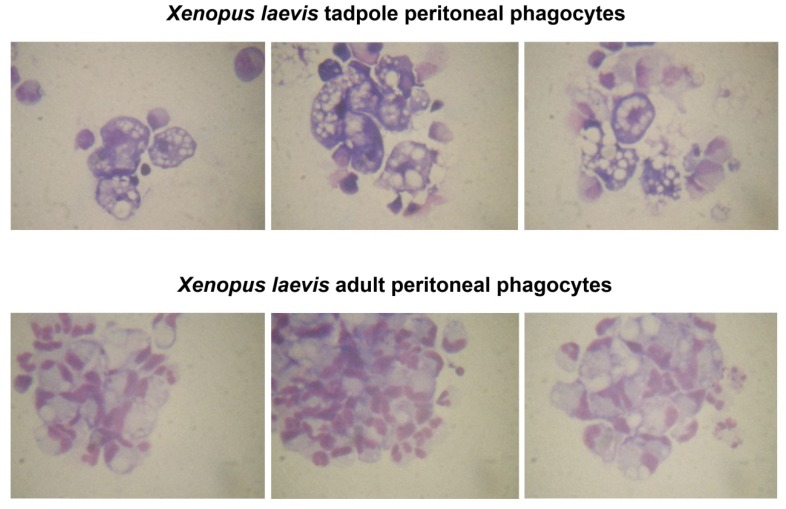
**Comparison of *Xenopus laevis* tadpole and adult peritoneal macrophages.** Peritoneal leukocytes were collected from frogs 3 days after elicitation with heat-killed *Escherichia coli*. Cells were seeded in and allowed to adhere to bottoms of 24 well plates for 3 hours. Non-adherent cells were removed, the adherent cells washed and cultured for further 4 days. Cells were cytospun onto glass slides and Giemsa stained according to standard protocol. Upper panels represent peritoneal macrophages collected from tadpoles and lower panels represent peritoneal macrophages derived from adult frogs.

It should be underlined that while *Xenopus* myelopoiesis is poorly understood, there are several fundamental changes that occur during development that could substantially influence macrophage-mediated immune responses. Notably, macrophage numbers and functions are dictated by development, where phagocyte numbers increase in late metamorphosis and subsequently decline [56]. It is becoming more apparent that cells of the macrophage lineage exhibit distinct activation states [57,58,59,60], where tissue-remodeling macrophages such as those predominating during *Xenopus* metamorphosis [56], exhibit poor antimicrobial potentials in mammals. Possibly, ranaviruses may rely on myeloid cells with effective phagocytic potentials (such as tissue remodeling macrophages) but poor antimicrobial/antiviral functions for persistence and dissemination. This might be reflected in the susceptibility and resistance to FV3 infections of tadpoles and adults, respectively. Interestingly, a subset of tadpole but not adult peritoneal phagocytes, seem to exhibit very vacuolar, enlarged morphology and distinct Geimsa staining patterns (Figure 2). 

**Figure 3:**
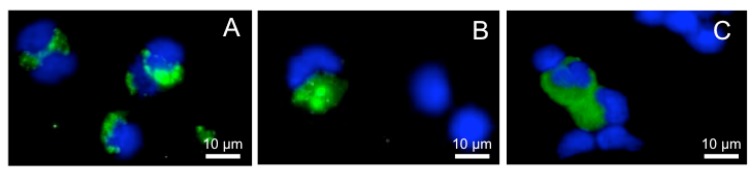
**Comparison of *Xenopus laevis* tadpole and adult peritoneal macrophages infected by FV3.** Peritoneal leukocytes were collected from one adult (**A**) or from 10 pooled pre-metamorphic tadpoles (**B **and **C**) 2 days after FV3 infection by intraperitoneal injection. Cells were fixed, stained with a rabbit anti-53R (green) and DNA dye Hoechst-33258 (blue), then mounted in anti-fade medium and visualized with a Leica DMIRB inverted fluorescence microscope.

Possibly these tadpole phagocytes represent tissue-remodeling alternatively polarized macrophages that would skew any inflammatory response to a viral infection. Furthermore, while MHC class II expression is present throughout frog development, MHC class I is not detected until the climax of metamorphosis [61]. Thus, immune surveillance and detection of any infected macrophages in pre-metamorphic animals would be considerably less efficient, underlining another potential ranaviral infection strategy. Consistent with this possibility, we recently found that larval PLs appear to be less resistant to FV3 infection than adult PLs (manuscript in review). This was determined by immunofluorescence microscopy using a rabbit polyclonal antibody specifically recognizing 53R, a putative 54.7-kDa myristoylated viral protein that is critical for FV3 replication. On average there were twice as many infected PLs stained by the anti-53R antibody in larvae than in adults. In addition, the wider staining pattern and higher signal intensity in larval PLs suggests that, compared to adult PLs, the viral load is higher (Figure 3 B, C and A, respectively).

We believe that further FV3 infection studies in this model organism will reveal, not only the biological mechanisms governing animal susceptibility and resistance, but also elucidate the underlining immune regulation strategies across developing animals.

## 4. Ranavirus Immune Evasion Mechanism

Ranaviruses possess large DNA genomes encoding between approximately 100 to 140 putative gene products. Although many of these genes encode proteins that play indispensible roles in viral replication and virion morphogenesis, it is hypothesized that other gene products encode proteins that expand host ranges or permit replication in certain cell types (*i.e.*, host range or efficiency functions) whereas still others counteract the host immune responses (viral immune evasion proteins). Gene products in the former category are absolutely required for replication *in vitro*, whereas products in the latter two categories may only be required in certain types of cells or for growth *in vivo*. While much remains to be learned about the functions of ranavirus gene products, recent studies have given new perspectives on the complexities of ranavirus-host interaction. 

### 4.1. Antiviral Properties of Eukaryotic PKR

Protein kinase R, also known as EIF2αK2 [62], was initially identified as a regulator of antiviral responses through protein synthesis studies in cell-free lysates from type I IFN and dsRNA treated cells [63]. This enzyme is part of a small family of protein kinases that respond to environmental stressors by regulating cellular protein synthesis through the phosphorylation of the alpha subunit of eukaryotic translational initiation factor 2 (eIF2α) [63]. Phosphorylation of the α subunit of eIF-2 blocks its ability to exchange GDP for GTP and results in the inhibition of protein synthesis at the level of initiation. The N-terminal domain of PKR functions as a steric inhibitor, and as a result PKR is predominantly found as an inactive monomeric state, unable to phosphorylate eIF2α [64]. However, in response to cellular activation and/or the presence of viral (or synthetic) dsRNA, PKR dimerizes and is activated by autophosphorylation. Activated PKR subsequently phosphorylates eIF-2α resulting in the inhibition of translation [65,66]. Since eIF2α is a key component of cellular translational machinery, its phosphorylation and hence inactivation establishes a state of cellular translation arrest, thus preventing cell proliferation and the synthesis of cellular and viral proteins. Furthermore, the PKR-mediated inactivation of this cellular translation factor may facilitate the apoptotic death of the infected cells [67], effectively eliminating the infecting agents. Despite this cellular checkpoint, many viruses have adopted mechanisms to subvert and/or overcome this translational block (reviewed in [68]).

### 4.2. Ranavirus PKR Inhibitors

Several members of the genus *Ranavirus* encode viral homologs that are pseudosubstrates (*i.e.*, decoy versions) of eIF-2α [69]. Viral homologs of this cellular translation factor have been cloned and sequenced from several ranaviruses including the Epizootic Haematopoietic Necrosis virus (EHNV) and the Rana catesbeiana virus (RCV) [69]. The nucleotide and putative amino acid sequences of these molecules share high identity with eIF2α homologs of poxviruses (designated K3L in vaccinia virus) and cellular eIF2α from species normally infected by these pathogens [69]. Sequence identity is marked within the N-terminal 90 amino acids of K3L, ranavirus vIF-2α, and cellular eIF-2α [69,72]. Surprisingly sequence analysis of our isolate of FV3 and that of a ranavirus isolated from softshell turtles (STIV) indicates that these isolates encode a truncated version of vIF-2α that is missing the N-terminal end of the gene, but retains the C-terminal two-thirds of the molecule [70]. To ascertain the function of vIF-2α, others and we have generated knock out mutants lacking this gene. The first attempt at determining the function of vIF-2α utilized *Ambystoma tigrinum* virus (ATV), a highly pathogenic ranavirus isolated from the tiger salamander [6]. Like EHNV and RCV, ATV encodes full-length homolog of eIF2α, designated vIF-2α [6]. The gene encoding vIF-2α (ORF57R) was deleted from ATV genome using homologous recombination and replaced with a selectable marker [6]. Compared to wild type ATV, the ATVΔ57R recombinant failed to inhibit the phosphorylation of host cell eIF2α, [6]. Moreover, the knock out virus was substantially more susceptible to PKR induction by the double stranded RNA analog, poly I:C [6]. Furthermore, when fathead minnow cells were infected with the wild type ATV, eIF2α phosphorylation was inhibited and correlated with the virus-induced degradation of the fish PKR-related enzyme, PKZ. Conversely, FHM cells infected with the Δ57R ATV displayed eIF2α phosphorylation, presumably due to the presence of a functional eIF-2α kinase [6]. 

In order to target putative FV3 virulence genes, our group has recently generated three recombinant viruses using improved site-specific-integration knock-out techniques [70]. One of these target genes was the truncated version of vIF-2α found in FV3 as we wished to see whether this protein, which retained the C-terminal end of the full-length molecule played any roles in viral replication. FV3ΔvIF2α replicated as well as wt FV3 in cell culture. The FV3ΔvIF2α replicated as well as wild type FV3 in cell culture. However, FV3ΔvIF2α exhibited impaired replication and lower mortality in infected *X. laevis* tadpoles as compared to wild type virus and a “knock in” control recombinant, expressing green fluorescent protein (FV3/GFP) [70]. This result suggests that the C-terminal end of the FV3 vIF-2α also plays an important role *in vivo*, underlining the importance of the FV3 vIF2α homolog in effective infections. 

The ranavirus *Rana* (*Lithobates*) *catesteiana* Virus Z (RCV-Z) also encodes a viral homolog of the host eIF2α, which shares identity with the cellular factor in the S1 helical and the C- terminal domains [71]. Moreover, RCV-Z vIF2α abrogates the toxic effects of the human and the zebrafish PKR enzymes in a yeast system [71]. This vIF2α appears to act as a pseudo-substrate and inhibits both the zebrafish and human PKR enzymes. Analysis of vIF-2α deletion constructs showed that both the N-terminal and the helical domains of the vIF2α were sufficient for the PKR inhibition, while the C-terminus of this viral protein was dispensable for these functions [71].

Notably, poxviruses also utilize viral homologs of eIF-2α that act as decoy substrates and prevent PKR phosphorylation. For example, the vaccinia virus gene product, K3L serves as a PKR pseudo-substrate and blocks PKR-mediated translational arrest. As described above, it seems that the viral pathogens of ectotherms such as those of the family *Iridoviridae* have also effectively adapted this strategy. It is surprising that FV3 vIF-2α is missing the N-terminal region homologous to vaccinia K3L and cellular eIF-2α and yet effectively synthesizes viral proteins in the face of a nearly complete inhibition of host protein synthesis. The ability of FV3 to maintain viral protein synthesis in the absence of a full-length vIF-2α gene product suggests that FV3 has an alternative way of blocking the activation of PKR, perhaps through a E3L-like protein. Further studies of the functions of ranavirus vIF-2α proteins will provide a broader and more thorough understanding of viral evasion strategies as well as the cellular pressures and responses dictating viral replication and infection strategies.

### 4.3. Ranavirus Virulence Determinants

As we have emphasized throughout this review, ranaviruses are complex pathogens that co-evolved with their ectothermic vertebrate hosts and as such, have undoubtedly developed numerous immune evasion and host persistence strategies. In fact, recent evidence suggests that ranaviruses can adapt and co-evolve relatively rapidly in new hosts [7]. Members of the ranavirus family possess large double stranded DNA genomes that contain as many as 100 open reading frames or putative genes, the products of most of which share no known viral or eukaryotic homologs. In a recent effort, investigators have employed a number of state-of-the art molecular techniques in order to gain a better understanding of the roles of these genes and gene-products in ranaviral infections and immune-evasion. 

FV3 serves as a great example of the complexity of this viral family, possessing a 105 kilobase pair genome and 98 putative open reading frames. Microarray analysis has recently been employed to map the temporal regulation of the FV3 genes through the course of infection and assign respective genes as immediate early (IE), delayed early (DE) and late (L) in accordance with their temporal involvement in infection progression [72]. This was achieved by first doing analysis of productive replication 2, 4 and 9 hours post infection and then utilizing cyclohexamide to block *de novo* protein synthesis and confirm the IE genes [72]. This work outlined the presence of some 33 IE and 22 DE genes, with putative roles in transcriptional regulation and DNA/ RNA synthesis as well 36 L genes, thought to play roles in DNA packaging and virion assembly. A further 7 genes could not be classified using these methods, and await further characterization using different approaches, perhaps using distinct cell types. Five of these FV3 genes were subsequently selected for anti-sense morpholino and siRNA knockdown studies [73]. By these means two IE genes, 46K and 32R were shown to be indispensable to *in vitro* FV3 replication in a fish cell line (FHM) [73]. Furthermore, it was determined that the genes encoding the major capsid protein, a large subunit of the viral RNA Polymerase II and a viral DNA methyltransferase were also all essential to the viral replication and infectivity in FHM cultures [73].

## 5. Concluding Remarks

Much remains to be learned regarding ranavirus gene regulation, cell invasion, the virus life cycle and immune evasion strategies. It is clear from the information presented here that there are definite gaps that must be bridged between what is currently known about the immune responses to these viruses, the viral infection strategies and the specifics of the mechanisms by which these pathogens so efficiently infiltrate hosts and even cross species barriers. These infectious agents encode an unprecedented number of putative gene products, several of which represent not only potential virulence factors but also the means to better understand both immune evasion strategies and the immune functions being manipulated. Ultimately, the study of ranaviruses in the context of their host immune systems holds the promise of providing insight into the pressures governing the evolution of both the viral invasion strategies as well as the host immune countermeasures.
